# Evolutionary History of a Gene Controlling Brain Size

**DOI:** 10.1371/journal.pbio.0020134

**Published:** 2004-03-23

Biologists have long known that the African great apes (including the chimpanzee, bonobo, and gorilla) are our closest relatives, evolutionarily speaking. The recent release of the chimp draft genome sequence confirms this relationship at the nucleotide level, showing that human and chimp DNA is roughly 99% identical. Given the genetic similarity between human and nonhuman primates, the next big challenge is to identify those changes in the human genotype (the genetic complement of an organism) that generated the complex phenotype (the physical manifestation of gene expression) that distinguishes humans from the great apes. For example, modern humans have larger brains and a larger cerebral cortex than both nonhuman primates and their forebears, the early hominids. Elucidating the molecular mechanisms that account for this expansion will provide insight into brain evolution.[Fig pbio-0020134-g001]

**Figure pbio-0020134-g001:**
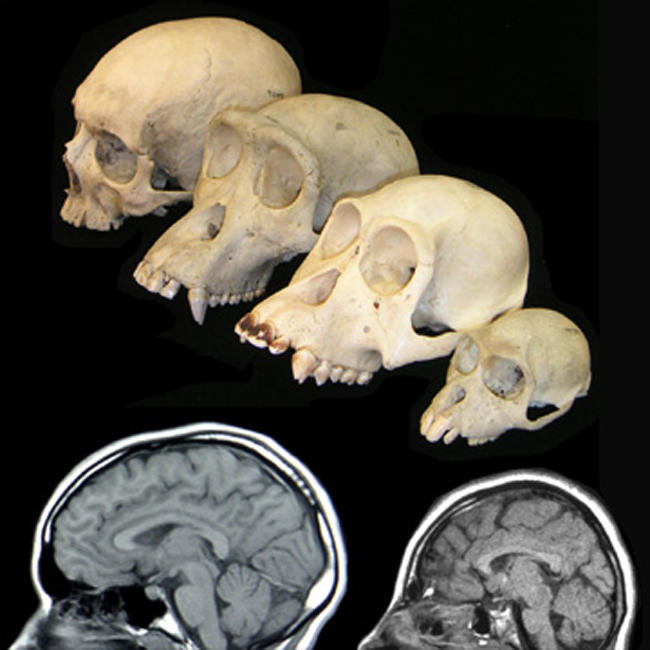
MRIs of a normal individual (bottom left) and a patient with microcephaly caused by an *ASPM* mutation (bottom right). Primate skulls provided courtesy of the Museum of Comparative Zoology, Harvard University

One way to figure out which genes are involved in a physiological process is to analyze mutations in the genotype that generate an abnormal phenotype. Such efforts are easier in the relatively rare instance that one gene affects a single trait. Mutations in the *ASPM* gene cause microencephaly, a rare incurable disorder characterized by an abnormally small cerebral cortex. Since the microencephalic brain is about the same size as the early hominid brain, researchers hypothesized that *ASPM*—whose normal function is unclear—may have been a target of natural selection in the expansion of the primate cerebral cortex. Last year, researchers showed that selective pressure on the *ASPM* gene correlated with increased human brain size over the past few million years, when humans and chimps diverged from their common ancestor. Now, Vladimir Larionov and colleagues report that the selective pressure began even earlier—as far back as 7–8 million years ago, when gorillas, chimps, and humans shared a common ancestor.

The researchers used a newly developed technology (called TAR-cloning) to extract specialized cloning agents in yeast (called yeast artificial chromosomes, or YACs) containing the entire *ASPM* gene, including promoter and intronic (noncoding) sequences, from chimpanzees, gorillas, orangutans, and rhesus macaques. They sequenced these YACs to determine the complete genomic sequence of the *ASPM* gene from each species. Next, they characterized sequence changes among these species, based on whether the resulting substitutions in amino acids produced changes in the ASPM protein, to determine how fast the protein was evolving. Larionov and colleagues found that different parts of the protein evolved at different rates, with the rapidly evolving sequences under positive selection (beneficial mutations were selected for, or retained) and the slowly evolving sequences under “purifying” selection (significant disruptions were jettisoned). Positive selection on genes is one important way to drive evolutionary change.

By reconstructing the evolutionary history of the *ASPM* gene, Larionov and colleagues show that the increase in human brain size—which began some 2–2.5 million years ago—happened millions of years after the gene underwent accelerated selective pressure. The *ASPM* gene, they conclude, likely plays a significant role in brain evolution. The next big challenge will be identifying the forces that preferentially acted on the human genotype to kick-start the process of brain expansion, forces that promise to shed light on what makes us human. New genomic technologies like TAR-cloning will likely accelerate this process.

